# Organizing Effects of Testosterone and Economic Behavior: Not Just Risk Taking

**DOI:** 10.1371/journal.pone.0029842

**Published:** 2011-12-29

**Authors:** Pablo Brañas-Garza, Aldo Rustichini

**Affiliations:** 1 GLOBE: Department of Economics, Universidad de Granada, Granada, Spain; 2 Department of Economics and Center for Cognitive Sciences, University of Minnesota, Minneapolis, Minnesota, United States of America; University of Akron, United States of America

## Abstract

Recent literature emphasizes the role that testosterone, as well as markers indicating early exposure to T and its organizing effect on the brain (such as the ratio of second to fourth finger, 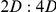), have on performance in financial markets. These results may suggest that the main effect of T, either circulating or in fetal exposure, on economic behavior occurs through the increased willingness to take risks. However, these findings indicate that traders with a low digit ratio are not only more profitable, but more able to survive in the long run, thus the effect might consist of more than just lower risk aversion. In addition, recent literature suggests a positive correlation between abstract reasoning ability and higher willingness to take risks. To test the two hypotheses of testosterone on performance in financial activities (effect on risk attitude versus a complex effect involving risk attitude and reasoning ability), we gather data on the three variables in a sample of 188 ethnically homogeneous college students (Caucasians). We measure a 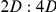 digit ratio, abstract reasoning ability with the Raven Progressive Matrices task, and risk attitude with choice among lotteries. Low digit ratio in men is associated with higher risk taking and higher scores in abstract reasoning ability when a combined measure of risk aversion over different tasks is used. This explains both the higher performance and higher survival rate observed in traders, as well as the observed correlation between abstract reasoning ability and risk taking. We also analyze how much of the total effect of digit ratio on risk attitude is direct, and how much is mediated. Mediation analysis shows that a substantial part of the effect of T on attitude to risk is mediated by abstract reasoning ability.

## Introduction

To understand human nature and socioeconomic behavior we need to understand not only the basic traits of individual personality separately, but also how they are related and interact with each other, and the biological basis of these traits and their connection. Two important traits that have been recently explored are reasoning ability and the willingness to take risks. In this paper we explore the connection between these two factors and the possible biological factors affecting them.

Among the biological factors influencing willingness to take risks, several studies have found both pre-natal and circulating testosterone (T) levels to be an important factor affecting behavior under uncertainty. The implications of these effects on real life, in addition to experimental behavior, may be large and important. For example, in [1] the level of daily profits in a sample of traders in the City of London was found to be positively correlated with the deviation from the median level of salivary T of each trader. Similarly [2], found the level of average profitability over a longer period to be negatively correlated with the ratio of the second to fourth finger (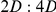 ratio). This ratio (see [3] for an introduction) is considered to be a marker of early (fetal) exposure to T.

A simple explanation of the link between T and performance in financial activities as found in [1] and [2] reduces higher profitability to the willingness to take risks. This account would suggest that on days when traders have a higher level of endogenous T relative to their own median level or a higher level of prenatal T exposure, traders' behavior is simply closer to risk neutrality, and they therefore choose, relative to other traders, portfolios with higher returns and higher variance. In the long run higher returns of the chosen portfolio ensure higher mean profits that we observe. This explanation interprets the correlation as a causal link from the level of T to attitude to risk. The explanation is appealing, but is unlikely to be complete. In fact, a higher variance in portfolio returns implies a higher variance in cumulated profits. If a lower bound on losses is imposed (for example, by the limit to total losses by the firm in the sample of traders in [2]), then a trader with higher propensity for risk should also be more likely to cease trading and exit the job. Thus, that account also predicts that traders who are more willing to take risks and earn higher returns should also experience a larger exit rate and shorter seniority on average. This is also what we should expect from theoretical investigations ([4], [5]) on the relationship between attitude to risk and survival in the market: everything else being equal (in particular, given the same inter-temporal preferences and beliefs, or information), risk-neutral traders are not those most likely to survive market selection. Instead, in [2] traders with a low digit ratio were also more likely to have higher seniority, indicating a higher probability of remaining on the job.

Willingness to take risks may be generally related to sensation seeking. Biological characteristics associated with this trait have been studied extensively, finding that T is one of the factors that has been associated with it ([6], [7]). In this paper we use the 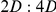 ratio as a measure in an experimental study. The specific link between the 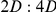 ratio and attitude to risk has been studied recently to test the hypothesis that lower digit ratios are associated with a higher willingness to take risks. The results are mixed (see [8] for a survey). An early study on 147 students found a positive correlation between digit ratio and risk aversion in an investment game (see [8]). The same result did not replicate in a sample of 125 ethnically heterogeneous subjects reported in the same study. Other studies that failed to replicate significance of the association are [9], [7], [10] and [11].

The sample in [9] was composed of 98 ethically heterogeneous subjects. [7] used a sample of 550 MBA students at the University of Chicago (381 male). The subjects were homogeneous in terms of age, cultural and educational background, and socioeconomic status. However, no precise information on ethnic background is provided in the text. They find that higher levels of circulating T is associated with lower risk aversion among women, but not among men. When they consider low concentrations of salivary T, the gender difference in risk aversion disappears; a result that suggests nonlinear effects of T on risk aversion regardless of gender. A similar relationship was also found between risk aversion and markers of prenatal T exposure. For example, the digit ratio was negatively correlated with the probability of pursing a career in finance. In a sample of 400 ethnically heterogeneous subjects [10], measure attitude to risk in bidding behavior. The task is a repeated two-bidder first-price sealed-bid auction with symmetric independent private values [11]. does not find an association between digit ratio and risk attitude (in a Holt and Laury task) in a sample of more than 200 subjects. A significant association between digit ratio and attitude to risk was found in [12] in a homogenous sample of 151 undergraduate students of both genders. Attitude to risk was measured by lottery choice in an Eckel–Grossman “50–50” [13] task [14]. find that the 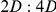 ratio predicts the amount of risk taken by 53 traders on a trading floor in the City of London but not their Sharpe ratios.

A possible explanation for the ability of low digit ratio traders to survive is that the biological factor represented by the marker affects, in addition to risk taking, the general ability of individuals to process information and perform cognitive tasks. Evidence that the 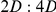 ratio affects some cognitive skills is surveyed in ([3], [15]), particularly in the areas of musical ability ([16]), spatial perception and cognition ([17], [18], [19], [20], [21]), verbal and numerical intelligence ([22]), memory recall ([23]), and the SNARC effect (Spatial Numerical Association of Response Codes) ([24]). Females affected by Congenital Adrenal Hyperplasia, a condition that exposes fetuses to high levels of androgens in the womb, scored higher on tests of spatial ability (i.e., Hidden Patterns, Card Rotations, and Mental Rotations: [25]). Recently, several studies ([26], [27], [28]), [29], [30]) have presented evidence that traits affecting economic behavior like risk aversion and impatience in choices among payments over time are correlated with several measures of cognitive skills. Specifically, for risk aversion, a finding which has been replicated ([27], [30]) is that a higher reasoning ability is broadly associated with higher willingness to take risks, particularly in the gain domain. Reasoning ability in the population may peak around risk neutrality ([27]). Recently, Manning and Fink [31] found a significant correlation of the 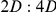 ratio with personality profiles in a sample of individuals in 23 countries. A result which is closely related to our study is their finding that a low per-country 2D∶4D ratio is associated with low risk aversion for the same country. They also find that a low 2D∶4D ratio per country is also associated with higher Gross Domestic Product. Possible explanations for this link may include the already noted higher risk tolerance associated with a low digit ratio, as well as a higher IQ in the population. Both explanations are consistent with the results we report below.

The hypothesis tested in the study we report is natural: a common biological factor (related to early exposure to T and reflected in the digit ratio) simultaneously influences reasoning ability and attitude to risk. We test this hypothesis in a simple experimental design where we gathered information on risk attitude, digit ratio, and reasoning ability in a sample of males and females. This analysis will allow us to test the relative size of the effects in addition to the simple existence of a correlation. We can also test the extent to which the effect of this biological factor directly influences risk attitude, and how much this effect works its way through reasoning ability. To determine this we will use simple mediation analysis, taking the digit ratio as the independent variable, the risk attitude as the dependent variable, and reasoning ability as the mediating variable.

## Results

### Summary statistics and gender differences

A summary description of the main variables of interest shows that our sample is, in all respects, typical. The digit ratio (DR) is around 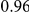 as is typical for a sexually heterogenous population. The ratio is sexually dimorphic, and significantly lower for men than for women. This is confirmed in our data (see Table 1).

**Table 1:** Summary statistics for 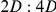.

	Observations	Mean	Standard Error	[95% CI]
All Subjects	188	0.958	0.0024	[0.953, 0.963]
Male	72	0.951	0.0035	[0.943, 0.957]
Female	116	0.963	0.0032	[0.956, 0.969]

Kruskal-Wallis equality-of-populations rank test: 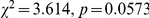. Two-sample Wilcoxon rank-sum (Mann-Whitney) test: 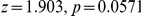.

The index of reasoning ability in our sample also has a typical distribution. Out of a total possible score of 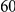, the mean score in the subjects' pool was 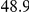, being higher for male subjects than for female subjects by around 3 points. The difference in this sample is statistically significant (see Table 2). There is no consensus on this very controversial topic, although the gender differences are usually recognized to be small or insignificant ([32], [33]). As we discuss in the conclusions, a possible explanation for this difference is the different motivation in the two genders. Size and significance of gender difference in reasoning ability is not very important for our purposes.

**Table 2:** Summary statistics for score in Raven's task (ReAb).

	Observations	Mean	Standard Error	[95% CI]
All Subjects	188	48.931	0.437	[48.076, 49.794]
Male	72	50.797	0.479	[49.840, 51.764]
Female	116	47.758	0.621	[46.527, 48.989]

The range in the sample was 12 to 60. Kruskal-Wallis equality-of-populations rank test: 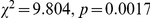. Two-sample Wilcoxon rank-sum (Mann-Whitney) test: 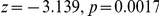.

As we mentioned above, we gathered two measures of risk aversion of the subjects: the lottery choice task and the Holt and Laury lottery choice task. Cronbach's 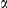 reliability index for the two combined sets of choices (16 items) is 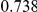,while the overall reliability index for the lottery choice task is 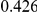. The set of choices in the Holt and Laury lottery choice task has a higher reliability coefficient: 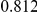. This is to be expected as 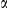 grows with the correlation among the scores in different tests, which in the case of the Holt and Laury lotteries are the choice for a fixed 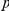. For subjects, consistency (hence higher correlation among those choices) is more likely when the choices are similar (same outcomes, different probabilities) and presented in an ordered fashion (increasing 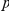).

We consider two measures of risk aversion in the analysis. The first is the number of safe choices in the lottery choice task,which we call the risk aversion measure (RA). The second is the sum of the safe choices in the two combined choice tasks. We call this variable the combined risk aversion measure (CRA). Summary statistics are reported in Table 3 (for the risk aversion measure) and Table 4 (for the combined risk aversion measure).

**Table 3:** Summary statistics for risk aversion measure (RA).

	Observations	Mean	Standard Error	[95% CI]
All Subjects	188	4.755	0.101	[4.554, 4.956]
Male	72	4.347	0.174	[3.998, 4.659]
Female	116	5.008	0.119	[4.772, 5.244]

The range in the sample was 1 to 7. Kruskal-Wallis equality-of-populations rank test: 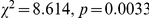. Two-sample Wilcoxon rank-sum (Mann-Whitney) test: 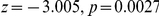.

**Table 4:** Summary statistics for the combined risk aversion measure (CRA).

	Observations	Mean	Standard Error	[95% CI]
All Subjects	188	9.444	0.225	[9.001, 9.892]
Male	72	8.944	0.344	[8.258, 9.630]
Female	116	9.758	0.294	[9.175,10.342]

The range in the sample was 3 to 16. Kruskal-Wallis equality-of-populations rank test: 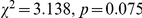. Two-sample Wilcoxon rank-sum (Mann-Whitney) test: 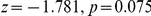.

Female subjects are significantly more risk averse than males, particularly in the risk aversion measure. This is consistent with the findings of a growing literature on the topic (see [34] for a survey of results).

The raw score in the Raven (Reasoning ability, ReAb) task is usually negatively skewed, and our data are typical in this respect. The score of risk aversion measures (CRA and RA) are approximately normal. Table 5 reports simple diagnostic tests of the distribution of the variables that are used in the analysis. They suggest the need for non linear transformations of the dependent variables (the Raven score) and independent variable (the digit ratio) in our analysis. We report this check, which does not alter the conclusions.

**Table 5:** Skewness/Kurtosis tests for Normality of the four variables: DR, ReAb, RA and CRA.

Variable	Pr(Skewness)	Pr(Kurtosis)	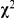	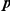 -value
Digit ratio (DR)	0.0057	0.112	9.11	0.017
Raven Score (ReAb)	0.000	0.000	73.47	0.000
Risk Aversion (RA)	0.0646	0.407	3.78	0.162
Combined Risk Aversion (CRA)	0.289	0.046	5.12	0.071

### Correlation Analysis

Two separate strands of the literature have identified a correlation between DR ([7]) and risk attitude on the one hand, and reasoning ability and risk attitude on the other ([27], [28], [29], [26]). In our data set we can test both potential relations, as well as the relation between digit ratio and reasoning ability.

The correlation coefficients and their significance are reported in Table 6 for the CRA measure and Table 7 for the RA measure. For male subjects, the results confirm the finding in ([27], [28] and [29]) of a negative correlation between reasoning ability and both measures of risk aversion (RA and CRA); and (consistently with the finding in [7]) show that subjects with a lower digit ratio are more willing to take risks. The correlation between DR and risk aversion, and the correlation between reasoning ability and risk aversion do not necessarily imply any correlation between DR and reasoning ability: we add to the findings reported in the literature a negative and significant correlation between digit ratio and reasoning ability in male subjects. The size of this latter correlation is also similar to the other two.

**Table 6:** Correlation analysis of Reasoning Ability (ReAb, measured by the Raven's score), Combined Risk Aversion measure and Digit Ratio.

	DR and ReAb		DR and CRA		CRA and ReAb	
All Subjects	−0.074		0.0106		−0.179	**
	*(0.308)*		*(0.885)*		*(0.013)*	
Male	−0.2629	**	0.240	**	−0.266	**
	*(0.0247)*		*(0.040)*		*(0.021)*	
Female	−0.0465		−0.145		−0.111	
	*(0.620)*		*(0.119)*		*(0.231)*	

The *, **, *** denote significance (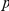-value) at the 1%, 5% and 10% level respectively. The entries indicate correlation coefficient, 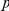-value is reported in parenthesis.

**Table 7:** Correlation analysis of Reasoning Ability (measured by the Raven's score), Risk Aversion measure and Digit Ratio.

	DR and RA		RA and ReAb	
All Subjects	−0.029		−0.1764	**
	*(0.692)*		*(0.014)*	
Male	0.1049		−0.2866	**
	*(0.376)*		*(0.013)*	
Female	−0.1881	**	−0.0595	
	*(0.043)*		*(0.524)*	

As we mentioned, the distribution of the Raven's score is skewed. The skewness in the distribution may be corrected with a Box Cox transform. If we do so, the sign of the correlation is unchanged, and its significance improves. For example, the significance of the correlation between the Box Cox transform of the Raven and digit ratio in males is 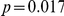.

The DR is sexually dimorphic, and we have found a strong correlation among males with both reasoning ability and risk aversion. It is natural to wonder whether the gender difference in the latter two variables is fully explained by the difference in digit ratio. The regressions of the reasoning ability score and the risk measures on the gender variables, the digit ratios, and the interaction between the two show (Table 8) that this is not the case.

**Table 8:** Regressions of Reasoning Ability (ReAb, measured by Raven's score, in the first column) and the two risk aversion measures (Combined RA and RA, second and third column respectively) on several regressors.

	ReAb	CRA	RA
	b/*p*-value	b/*p*-value	b/*p*-value
Male	0.468^***^	−0.140	−0.346^**^
	*(0.002)*	*(0.377)*	*(0.026)*
Digit ratio	0.050^*^	−0.139	−0.163^*^
	*(0.568)*	*(0.117)*	*(0.058)*
Male 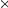 Digit ratio	−0.250	0.331^**^	0.202
	*(0.105)*	*(0.038)*	*(0.204)*
Raven score		−0.098	−0.046
		*(0.233)*	*(0.566)*
Male 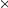 Raven Score		−0.203	−0.381
		*(0.290)*	*(0.043)*
constant	−0.199^**^	0.114	0.208^**^
	*(0.030)*	*(0.224)*	*(0.023)*
N	188	188	188

All variables (except male) are normalized to have zero mean and unit standard deviation.

For reasoning ability, the variable *Male* (which is equal to one for male subjects) is significant even when the digit ratio is among the independent variables. For the RA measure, the variable *Male* and the interaction of the gender variable with the reasoning ability score are both significant. These findings suggests a complex relation between our three main variables and gender, which we will now try to unravel.

### Mediation Analysis

Our correlation analysis shows that two factors may potentially affect attitude to risk: one is described by a biological marker, the DR, while the other is the reasoning ability of the individual. If we want to compare the relative strength and significance of the two, we can run a regression of our measure of risk attitude on both variables. To make the size of the estimated coefficients comparable, we first normalize all the variables to mean zero and unit standard deviation. The result of the regression of the RA measure on these normalized variables is reported in the last column of Table 9 for male subjects and Table 10 for female subjects. In the regression for male subjects, the coefficient of the DR is 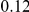 (*p*-value = 0.397) and the coefficient of reasoning ability is 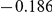 (*p*-value = 0.041). For female subjects, we observe a different pattern: the coefficient of the DR is 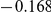 (*p*-value = 0.043) and the coefficient of reasoning ability is 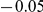 (*p*-value = 0.620).

**Table 9:** Mediation Analysis of the effect of digit ratio on risk attitude in male subjects.

	Risk Aversion on DR	ReAb on DR	RA on DR and ReAb
	b/*p*-value	b/*p*-value	b/*p*-value
Digit ratio	0.120	−0.186^**^	0.04
	*(0.397)*	*(0.041)*	*(0.775)*
Raven's score			−0.427^**^
			*(0.022)*
constant	−0.257^**^	0.27^***^	−0.142
	*(0.05)*	*(0.002)*	*(0.294)*
N	72	72	72

The risk attitude measure is the Risk Aversion measure. The mediating variable is Reasoning ability, (ReAb) measured by Raven's score. All variables are normalized to mean zero and unit standard deviation.

**Table 10:** Mediation Analysis of the effect of digit ratio on risk attitude in female subjects.

	Risk Aversion on DR	ReAb on DR	RA on DR and ReAb
	b/*p*-value	b/*p*-value	b/*p*-value
Digit ratio	−0.168^**^	0.050	−0.165^**^
	*(0.043)*	*(0.620)*	*(0.047)*
Raven's score			−0.046
			*(0.546)*
constant	0.218^**^	−0.199^*^	0.208^**^
	*(0.012)*	*(0.060)*	*(0.018)*
N	116	116	116

See table 9 for details.

This simple model does not consider the possibility that the effect of the DR may occur both directly on risk attitude and indirectly through its effect on reasoning ability. We have reason to consider this hypothesis in view of the correlations reported in tables 6 and 7, which suggest that reasoning ability might act as a mediating variable between the biological factors represented by the digit ratio and risk aversion. It is also reasonable to take the biological factors as an independent variable that determines the others since they are fixed at birth.

**Figure 1:**
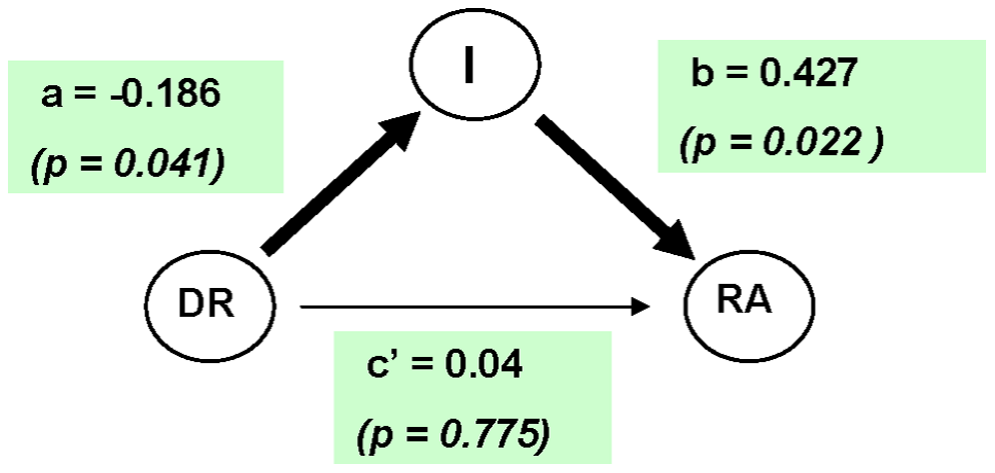
Mediation analysis of the effect of digit ratio (DR), direct and mediated by Reasoning ability (ReAb), on Risk Aversion (RA, risk aversion measure) in male subjects. The coefficients and 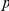-values reported in the figure refer to the Raven's score as measure of Reasoning ability, and number of safer choices made in the lottery choice task: see Table 9 for details.

A systematic way of testing this hypothesis is through mediation analysis ([35], [36], [37], [38], [39]). The model we consider is simple mediation. The model is illustrated in Figure 1 which reports the estimates for male subjects. In this figure, the three circles represent the variables we consider, while the arrows indicate the direction of causality. The DR may influence risk aversion directly, or through the path passing through reasoning ability. Mediation analysis tries to determine the size of the direct and mediated effect.

Simple mediation analysis is performed in two steps. In the first step we estimate three regressions among an independent variable (DR, digit ratio in our case), a dependent variable (RA, risk aversion in our case), and a mediating variable (ReAb as measured by Raven's score). The mediating variable is influenced (in a causal sense) by the independent variable and in turn influences the dependent variable. The three regressions are:
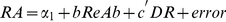
(1)

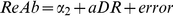
(2)

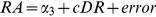
(3)


The regression of risk aversion on reasoning ability (ReAb) and the digit ratio gives an estimate of the coefficients 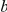 and 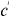 (equation 1). The results of this estimate are reported in the last column of Table 9 for the case of male subjects and the RA measure. We then estimate coefficient 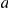 separately by regressing our measure of reasoning ability on digit ratio (equation 2). The results are reported in the second column of Table 9. The product of the two coefficients 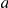 (which estimates the effect of DR on reasoning ability) and 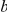 (which estimates the effect of reasoning ability on risk aversion) gives the size of the indirect (mediated by reasoning ability) effect of the digit ratio on risk attitude. Coefficient 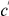 estimates the direct effect of the digit ratio on risk aversion when we are also conditioning on the indirect effect from reasoning ability.

In the second step we estimate the significance of the direct and indirect effects. The ratio of the product 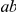 (indirect effect) over the sum 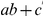 (direct and indirect effect) gives a measure of the fraction of the effect mediated by reasoning ability. The Sobel-Goodman (SG) statistic ([40], [41]) tests the hypothesis that the product 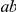 of the estimated coefficients is different from 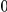. The Sobel statistic is derived by approximating the standard error of the product of the estimated 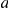 and 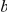 using a Taylor's series expansion, and is correct under the assumption that the product is normally distributed as the sample size becomes large. This assumption, however, is unlikely to hold when the null hypothesis that 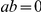 is not true. Recently [42], (see also [43]) proposed estimating asymmetric confidence intervals of the product 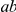 using bootstrapping methods. In our case the two methods give very similar estimates of the confidence intervals and the significance of the product.

Table 9 reports the regressions necessary for the case of male subjects for the RA measure. The percentage of the total effect that is mediated by reasoning ability is 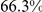 (Sobel-Goodman test: 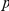-value = 0.095).

Table 10 reports the same results in the case of female subjects. The percentage of the total effect that is mediated by reasoning ability is 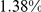 (Sobel-Goodman test: 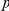-value = 0.812).

The pattern is consistent with the one we have just seen in the case of the CRA measure. Table 11 reports the result for male subjects, and Table 12 for females.

**Table 11:** Mediation Analysis of the effect of digit ratio on risk attitude in male subjects.

	CRA on DR	ReAb on DR	CRA on DR and ReAb
	b/*p*-value	b/*p*-value	b/*p*-value
Digit ratio	0.211^*^	−0.187^**^	0.191
	*(0.094)*	*(0.041)*	*(0.124)*
Raven's score			−0.293^*^
			*(0.076)*
constant	−0.116	0.270^***^	−0.036
	*(0.311)*	*(0.001)*	*(0.762)*
N	72	72	72

The risk attitude measure is the Combined Risk Aversion measure. The mediating variable is Reasoning ability, (ReAb) measured by Raven's score. All variables are normalized to mean zero and unit standard deviation.

**Table 12:** Mediation Analysis of the effect of digit ratio on risk attitude in female subjects.

	CRA on DR	ReAb on DR	CRA on DR and ReAb
	b/*p*-value	b/*p*-value	b/*p*-value
Digit ratio	−0.144	0.050	−0.139
	*(0.119)*	*(0.620)*	*(0.132)*
ReAb (Raven's score)			−0.098
			*(0.251)*
constant	0.133	−0.199	0.114
	*(0.165)*	*(0.060)*	*(0.242)*
N	116	116	116

See Table 11 for details.

We may conclude that a substantial part of the effect of digit ratio on risk attitude is mediated in male subjects by its effect on reasoning ability. As we should expect, mediation does not occur for female subjects because there is no effect from DR on risk aversion in the first place, as tables 6 and 7 have shown.

## Discussion

We have reported four main findings. Let us first consider male subjects. Our first finding is that the 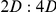 digit ratio (DR) is significantly correlated in male subjects with both reasoning ability and attitude to risk aversion. The correlation of the DR is negative and significant for reasoning ability. If we use our combined measure of risk aversion, the correlation is positive and significant for risk aversion. For both reasoning ability and combined risk aversion the correlation is around 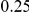 in size, and significant at better than a 5 per cent level (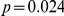 for reasoning ability and 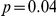 for the combined measure of risk aversion). Reasoning ability and combined risk aversion are also negatively correlated in male subjects, that is, a higher reasoning ability is associated with a lower willingness to take risks. The size effect is 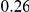 (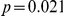).

Let us now turn to female subjects. Our second finding is that these correlations are not significant in females. In the case of the RA measure derived from lottery choice, the correlation between the digit ratio and risk attitude is significant but *negative*, that is, a higher ratio is associated with a smaller risk aversion; the opposite of what we found in male subjects for the combined measure of risk aversion.

The raw correlation results are easier to interpret in light of simple mediation analysis if we take the DR as the independent variable, risk aversion as the dependent variable, and reasoning ability as the mediating variable. The analysis attempted to determine how much of the total effect of biological factors expressed in the DR affect the risk attitude directly, and how much indirectly through the effect on reasoning ability.

Our third finding is that, in male subjects, a substantial part of the effect of the DR on risk attitude is mediated by the effect on reasoning ability. The precise extent of this effect varies depending on the measure of risk attitude that is being used, and is between 30 and 70 per cent. The final and fourth finding is that this mediation effect is absent in females. In summary, it appears that the mechanism of transmission between biological features represented by the DR marker is substantially different in males and females. This conclusion is supported by our analysis of the effect (in the entire sample) of the digit ratio and interaction with gender on reasoning ability and risk aversion (Table 8).

These findings help to explain one of the initial puzzles: how do low digit ratio traders survive ([2]) in the market? Our results indicate that individuals with a low digit ratio are at the same time more inclined to take risks and more effective in processing information. This joint effect would contribute to explaining the higher profitability (in addition to the effect on risk attitude) as well as the ability to survive due to a better discrimination in the choice of investment strategies.

However, a word of caution is necessary. We measured reasoning ability with a test, and performance in a test is the joint outcome of at least two factors: skill and effort. A high score on a reasoning ability test may be due to differences in motivation among subjects; a factor that can in part explain the observed correlation between the digit ratio and reasoning ability. For example, if male subjects with a lower digit ratio are also more sensitive to inter-personal comparisons of outcomes, this motivation would systematically affect the effort component, thus making the observed performance of these subjects systematically better even in the absence of differences in intellectual skill. In our experiments, the score on the Raven test was privately announced to single subjects one month after the test, so it is unlikely that the motivation to excel over others in public played a significant role. This feature of the experimental design does not yet preclude the possibility that an internal motivation, independently of the observability of the relative outcome, played some role, although overall the effect is likely to be modest. Separating the effect of the biological factors represented by the digit ratio on skill and motivation seems to be the next step in the research agenda.

## Materials and Methods

### Sample

A total of 189 ethnically homogeneous (Caucasians) subjects participated in the experiment. One subject of Asian ethnicity was excluded to ensure the homogeneity of the pool, so a total final number of 188 subjects was used in the analysis. Several papers (see for example [44] and [45]) demonstrate that the 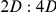 ratio varies substantially among different ethnic groups; hence it is important that all subjects belong to the same ethnicity to ensure that the relation between the digit ratio and variables of interest are not confounded by differences in the composition of the sample. 72 subjects were male. The average age of the subjects was 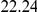 years old (standard deviation 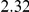, mean of 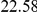 for male, range of 19 to 31 years).

### Reasoning ability and risk aversion tests

Reasoning ability was measured with Raven's Progressive Matrices. The test consists of 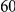 multiple choice questions originally developed by John C. Raven [46]. In each test item, a candidate is asked to identify the missing item required to complete a larger pattern. The final score is a measure of abstract reasoning ability and fluid intelligence, which is an ability that does not rely on knowledge or skill acquired from experience (as opposed to crystallized intelligence, see [47]).

Attitude to risk was measured through observed choice between random payments, or lotteries. Subjects faced two sets of choice tasks in which they had to choose between two lotteries. In the first, one of the lotteries (called here “safer”) had an expected value smaller or equal to the other one, but smaller variance. For example, subjects were asked to choose between a payment of 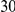 euros for sure or a payment of 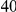 euros with a probability of 80 per cent. One of the lotteries had a loss as a possible outcome. In four out of seven choices, the safer option was a certain amount. Table 13 reports the lotteries given in this task, which we call the lottery choice task. Payment was hypothetical, i.e. the subjects were asked to state what they would choose if the lotteries paid real money. Experimental testing shows that the provision of hypothetical payments does not affect the mean of the measured variables but rather the variance (see [48]).

**Table 13:** Lotteries presented in the lottery choice task.

Safer lottery	Riskier lottery
(30, 1)	(45, 0.8, 0)
(1000, 1)	(2000, 0.5, 0)
(100, .25, 0)	(130, .2, 0)
(3000, 0.02, 0)	(6000, 0.01, 0)
(50, 1)	(50, 3/6, 200, 1/6, 0)
(50, 3/6, 0)	(200, 1/6, 0)
(0, 1)	(1500, 0.5, −1000)

The lottery in the left column has lower or equal mean as the lottery in the left column, but the variance of the lottery in the right is higher.

The second task is a lottery choice task used by Holt and Laury ([49], [50]). Subjects faced a set of nine choices between two lotteries. The notation 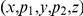 describes the lottery giving the amount 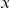 (in euros) with a probability 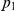, 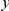 with a probability 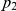, and 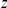 with the complementary probability 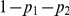. The set of outcomes of the two lotteries was the same in every choice: one lottery was 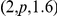 (a “safer” lottery”), and the other was 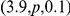. Probability 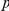 ranged from 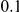 to 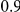 in increments of 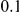, giving the subjects nine options overall. As 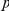 increases, the difference in expected utility for an expected utility decision maker between the first and second lottery decreases from a positive to negative value. The number of times a subject chooses the first lottery is the measure of his risk aversion provided by this task. We will refer to this task as the Holt and Laury lottery choice task. The experiment was run using z-tree software ([51]).

### 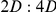 data

Data on the 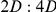 ratio (DR) were collected as outlined in [52]. We first obtained a photocopy of the ventral surface of the right hand of the subject. Only a photocopy of the right hand was obtained, as this is the hand that is widely used for study of the 2D∶4D digit ratio. Subjects were asked to straighten their fingers and gently press the hand on the photocopier's plate. The quality of the photocopy was checked. Later, two landmarks were marked at the crease at the base of the finger proximal to the palm and the tip of the finger, and then a measure of the length of the two fingers was obtained with a ruler.

Researchers collecting the finger length did not know the choices made by the subjects in the decision problems nor their performance in the reasoning task. They also did not have gender or general information on the subject. The choice and reasoning tasks were administered on computers, and the researchers running this section of the experiment did not know the finger length of the subjects.
